# The immunomodulatory effects of GLP-1 receptor agonists in neurogenerative diseases and ischemic stroke treatment

**DOI:** 10.3389/fimmu.2025.1525623

**Published:** 2025-03-11

**Authors:** Haohui Sun, Yue Hao, Hao Liu, Feng Gao

**Affiliations:** School of Basic Medical Science, School of Medicine, Ningbo University, Ningbo, Zhejiang, China

**Keywords:** glucagon-like peptide-1 receptor agonists, Treg cells, microglia, IL-10, ischemic stroke

## Abstract

Glucagon-like peptide-1 (GLP-1) receptor is widely distributed in the digestive system, cardiovascular system, adipose tissue and central nervous system. Numerous GLP-1 receptor-targeting drugs have been investigated in clinical studies for various indications, including type 2 diabetes and obesity (accounts for 70% of the total studies), non-alcoholic steatohepatitis, Alzheimer's disease, and Parkinson's disease. This review presented fundamental information regarding two categories of GLP-1 receptor agonists (GLP-1RAs): peptide-based and small molecule compounds, and elaborated their potential neuroprotective effects by inhibiting neuroinflammation, reducing neuronal apoptosis, and ultimately improving cognitive function in various neurodegenerative diseases. As a new hypoglycemic drug, GLP-1RA has a unique role in reducing the concurrent risk of stroke in T2D patients. Given the infiltration of various peripheral immune cells into brain tissue, particularly in the areas surrounding the infarct lesion, we further investigated the potential immune regulatory mechanisms. GLP-1RA could not only facilitate the M2 polarization of microglia through both direct and indirect pathways, but also modulate the quantity and function of T cell subtypes, including CD4, CD8, and regulatory T cells, resulting into the inhibition of inflammatory responses and the promotion of neuronal regeneration through interleukin-10 secretion. Therefore, we believe that the "Tregs-microglia-neuron/neural precursor cells" axis is instrumental in mediating immune suppression and neuroprotection in the context of ischemic stroke. Given the benefits of rapid diffusion, favorable blood-brain barrier permeability and versatile administration routes, these small molecule compounds will be one of the important candidates of GLP-1RA. We look forward to the further clinical evidence of small molecule GLP-1RA intervention in ischemic stroke or T2D complicated by ischemic stroke.

## 1 The neuroprotection of glucagon like peptide-1 receptor agonists in neurodegenerative and cerebrovascular disorders

Glucagon like peptide-1 (GLP-1) is a peptide called enteropancreatin secreted by small intestinal L cells. Physiologically, it can bind to GLP-1 receptor on pancreatic beta cells through endocrine, activate adenylate cyclase, increase intracellular cAMP levels, promote glycolysis and ATP production, and enhance glucose-stimulated insulin secretion. The direct regulation of GLP-1 on insulin expression is significant for type 2 diabetes (T2D) patients with simple glucose tolerance but normal insulin secretion (1). It can avoid adverse reactions and potential risk of pancreatic gland degeneration caused by direct insulin treatment (2). It is worth noting that GLP-1 can easily spread to the central system and exert neuroprotective and neuropathic actions in various neurodegenerative and cerebrovascular disorders (Alzheimer’s disease (AD), Parkinson’s disease (PD), stroke, etc.) through biological signal transduction (3–5). However, due to the short half-life of endogenous GLP-1 in the body (only about 2 minutes), it will be rapidly degraded by dipeptidyl peptidase-4 (DPP-4) and neutral endopeptidase. Therefore, GLP-1 derivatives with medium to long acting properties have become the main GLP-1RA for clinical applications, such as exendin-4, exenatide, liraglutide, lixisenatide, semaglutide, etc. (6–8). These peptide-based GLP-1RA have been widely studied globally for improving cognitive dysfunction in patients with neurodegenerative diseases (AD and PD) (ID: NCT03456687; NCT01255163; NCT03439943; NCT03659682; NCT01843075; NCT02953665) (9–12).

## 2 Peptide-based GLP-1 derivatives and small molecule compounds of GLP-1RA

**Figure 1:**
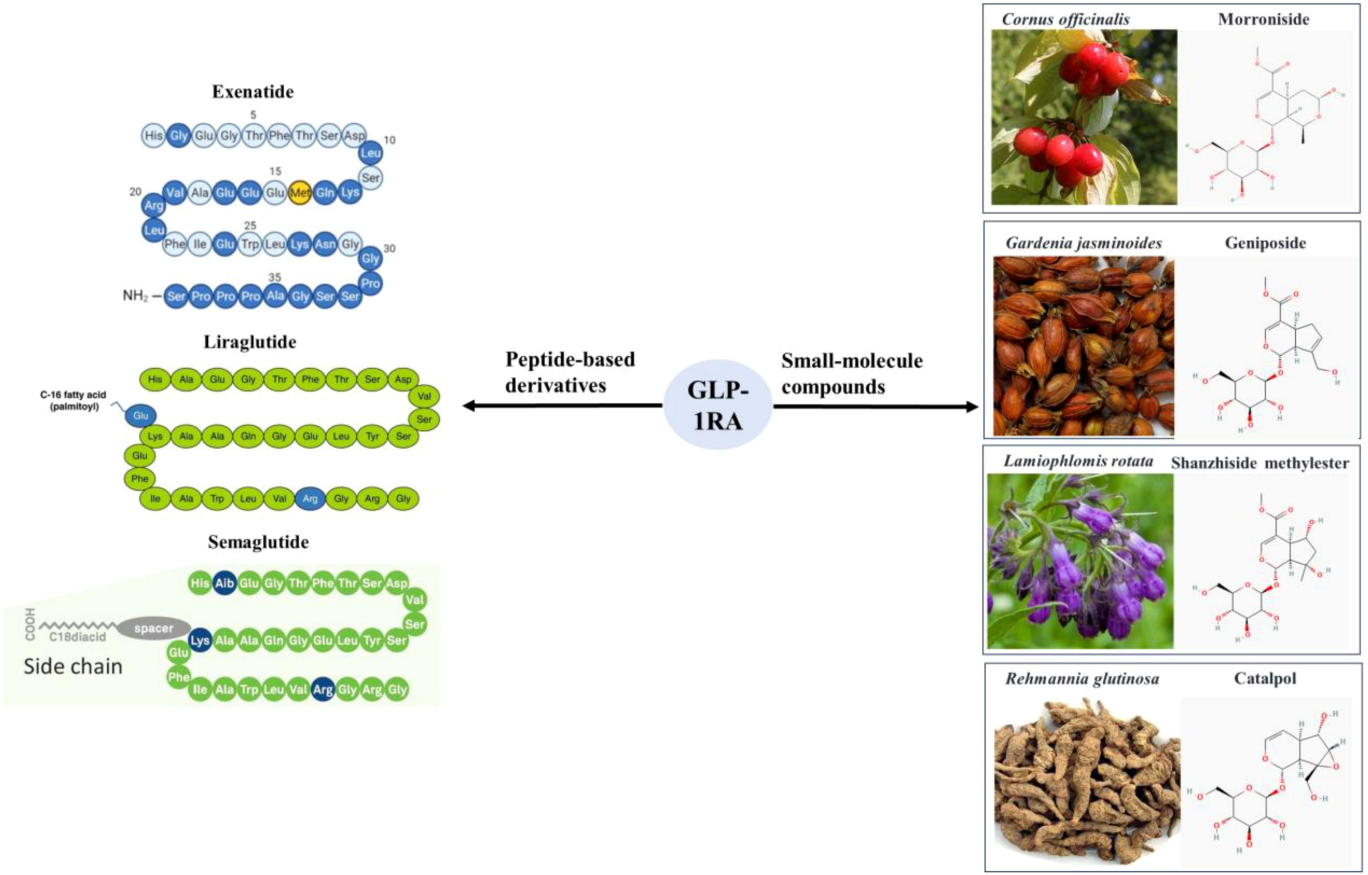
Two main categories of GLP-1 receptor agonists, small molecule compounds and peptide-based GLP-1 derivatives.

There are two main categories of GLP-1 receptor agonists, small molecule compounds and peptide-based GLP-1 derivatives as shown in **Figure 1**, including exenatide, liraglutide, dulaglutide, etc. Many literatures proved that GLP-1 derivatives performed neuroprotective effects and inhibited neuronal apoptosis (13–15). It was reported that cerebral expression of GLP-1 receptor protein reduced following subarachnoid hemorrhage (SAH), but markedly increased by liraglutide (15). Liraglutide also alleviated SAH-induced early brain injury and inflammation. Exendin-4 not only reduced the infarct size of ischemic stroke, improved the neurologic deficit scores in ischemia/reperfusion-induced rats, but also reduced the potential rtPA-induced hemorrhagic risk through the Wnt/β-catenin signaling pathway (16).

There are also a type of non-peptide small molecules, which are mainly extracted from herbal medicine, such as *Cornus officinalis*, *Gardenia jasminoides*, *Rehmannia glutinosa*, *Lamiophlomis rotata*, and *Strychnos nuzi* (17–20). These small molecule compounds mainly belong to iridoid glycosides, including geniposide, Shanzhiside methylester (SM), 8-O-acetyl-SM, morroniside, catalpol, genipin methyl ether and so on (18, 21). These GLP-1RA could be used to treat diabetes and its complicated obesity, fatty liver, hypertension and various cardiovascular diseases (22). In addition, accumulating studies demonstrated that GLP-1RA performed neuroprotective effects in a variety of neurodegenerative diseases (mainly AD, PD and stroke) (23–25).

Even for patients without complications of diabetes, their cognitive functions were still significantly improved (26, 27). The notable therapeutic impact of peptide-based GLP-1 derivatives in clinical neurodegenerative disorders had fostered enthusiasm among researchers to develop small molecule GLP-1RA. It was reported that small molecule GLP-1RA could reduce chronic neuropathic pain and exert neuroprotective effects by improving synaptic plasticity and ameliorating neuronal death (28, 29). GLP-1RA inhibited inflammatory responses in chronic pain through the GLP-1R/PI3K/Akt/ERK-1/2/IL-10 signaling pathway and reduced the release of inflammatory factors such as IL-6, IL-1, tumor necrosis factor alpha (TNF-α) (30). In addition, Wang Y.X. also found that the analgesic effects of various small molecule GLP-1RA such as lemairamin mainly relied on their promotion of IL-10 secretion and subsequent IL-10R/β endorphin signaling pathways, which effectively alleviated bone cancer pain and neuropathic pain (31–33). In these studies related to analgesia, shanzhizhi methyl ester performed a dose-dependent and persistent (>4 hours) effects against allodynia (17). In rodent models of neurodegenerative diseases, a series of iridoid glycosides represented by geniposide and mononucleoside could effectively resist cellular oxidative damage and counteract neuronal apoptosis (19, 28). In summary, as a promising class of small molecule GLP-1RA, iridoid glycosides represent a potential therapeutic avenue for the management of neurodegenerative disorders.

## 3 The neuroprotection of GLP-1RA in experimental stroke

**Figure 2:**
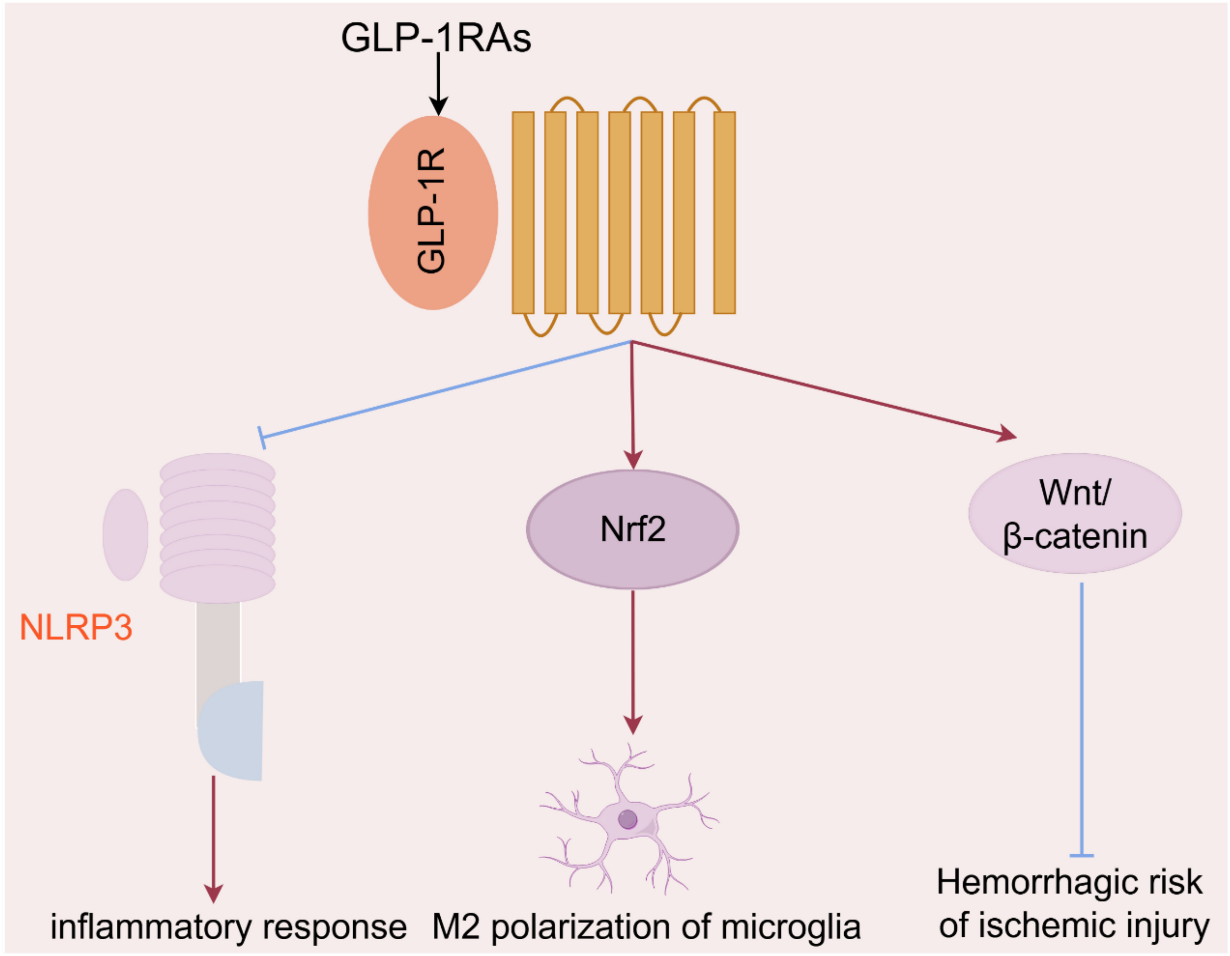
The principal mechanisms of GLP-1RAs in the regulation of neurological function in ischemic stroke.

Many literatures indicated that GLP-1RA were also considered as the latent intervention strategy against ischemic stroke to improve the pathological symptoms and prognosis. The principal neurological function regulated by GLP-1RA could be summarized as follows (**Figure 2**): (1) GLP-1RA liraglutide and exendin-4 effectively inhibited neuronal apoptosis (15), and ultimately improved early brain injury by suppressing NLRP3 inflammasome and inflammatory reaction in microglia (34–36); (2) Exendin-4 meliorated vascular permeability by inhibiting blood-brain barrier breakdown and leakage (37), and reduced the hemorrhagic risk of ischemic insults via the Wnt/beta-catenin signaling pathway (16). Besides, exendin-4 also dilated cerebral arterioles, raised cerebral blood flow and finally reversed the cerebral ischemia (38). (3) GLP-1RA (e.g. morroniside, exendin-4) promoted M2 polarization in microglia by upregulating nuclear factor erythroid 2-related factor 2 (28), and further improving synapse growth and neuronal regeneration via the cAMP/PKA pathway (39), and finally reduced cognitive and memory impairment induced by stroke (40). GLP-1RA appeared to exert neuroprotective effects primarily by regulating microglia, which were the most significant immune cells in the central nervous system (41).

## 4 GLP-1RA reduced the risk of stroke in T2D patients clinically

As a new type of hypoglycemic drug, GLP-RAs are currently mainly used in clinical practice to evaluate the prognosis of various complications in T2D patients, such as stroke, kidney injury, fatty liver, and cardiovascular disease (42, 43). Compared with other new hypoglycemic drugs such as SGLT2 and DPP-4, GLP-1RA have a unique preventive effect in reducing the risk of stroke as shown in **Table 1** (43, 45). With the ageing of society, stroke has emerged as the leading cause of mortality and major cause of disability worldwide, greatly increasing the burden of human society (46). According to the degree of severity, stroke-related disability led to premature death with disability-adjusted life-years (DALYs) (47). There was a notable discrepancy in the proportion of deaths resulting from hemorrhagic stroke among different racial and ethnic groups. The proportion of deaths due to hemorrhagic stroke among middle-aged and older people (≥35 years) in the 1990s was 38% for Asians, 32% for Hispanics, 24% for blacks, and 18% for whites (47, 48). The high rates of intracerebral hemorrhage (ICH) in Asian populations have been attributed to the high prevalence of hypertension, smoking and metabolic syndrome (49). Using Caucasians as a reference, the adjusted relative risk (RR[95%CI]) of ICH in Asians was 1.6[1.1-2.3] (P=0.01) (50, 51). The mortality rate increased by 32% from 1990 to 2019 (52). In 2019, there were 2.2 million deaths and 45.9 million DALYs after stroke insults in China (53). By 2050, the burden of stroke in individuals (aged ≥65 years) will increase significantly: 104.7% in incidence, 218.5% in prevalence, 100.0% in mortality, and 58.9% in DALYs (54).

**Table 1:** New hypoglycemic drugs reduced the risk of multiple complications in T2D patients.

Novel hypoglycemic drugs	Cardiovascular events (43, 44)	Nonfatal stroke (43)	Renal events (43, 45)	Fatty liver (42)
GLP-1RA	++	++	++	++
SGLT2i	++		+++	++
DPP-4i				

++ represent significance compared to placebo; +++ represent significance compared to other positive drug (++).

Many systematic reviews with network meta-analysis summarized the risk of noval hypoglycemic drugs on diabetic complications, including cardiovascular events, nonfatal stroke, renal composite outcomes and fatty liver (42–45). By using random-effects model, the network meta-analysis indicated that the risk profile of DPP-4i was same as placebo in all the outcomes (44). Distinctly, both SGLT-2i and GLP-1RA significantly decreased the risk of cardiovascular events (odds ratios (OR)[95%CI], GLP−1RA: 0.87[0.82-0.93]; SGLT−2i: 0.88[0.82-0.95]), and renal composite outcome (GLP−1RA: 0.86[0.78-0.94); SGLT−2i:0.59[0.52-0.67]) when compared to placebo (**Table 1**). In addition, only administration of GLP-1RA resulted into lower nonfatal strokes (OR, 0.88[0.77-0.99]) than those receiving placebo. P-rank scores confirmed that GLP-1RA reduced the risk of nonfatal stroke by 80.6%.

The similar viewpoints were also supported by other critical reviews and clinical trials (55–58), and inferred that GLP-1RA could be considered in patients at a high risk of, or with established cardiovascular diseases to improve the prognosis of cardiovascular events (especially nonfatal stroke), whilst SGLT2i could be recommended for patients with heart failure or chronic kidney diseases.

## 5 Immune dysfunction in ischemic stroke and potential immunomodulatory effects of GLP-1RA

### 5.1 The regulatory function of immune cells in the progression of ischemic stroke

The latest research shows that healthy brains do not lack immune cells. Normal cerebrospinal fluid contains about half a million immune cells, with T cells being the main type (about 50% CD4^+^ and 20% CD8^+^) (59). These cells interact bidirectionally with resident immune cells microglia and astrocytes, participating in antigen presentation by antigen-presenting cells, activation of T cells, and promotion of lymphatic system circulation in cerebral spinal fluid (60). In the pathological state of ischemic stroke, inflammatory signals released by dead neurons or cytokines and chemical inducers secreted by microglia under stress could drive circulating lymphocytes to recruit to the damaged brain (61, 62). Concurrently, the disruption of the blood-brain barrier results in a notable influx of immune cells into brain tissue. A substantial body of evidence suggests that both peripheral innate immune cells (such as neutrophils, macrophages, and natural killer cells) and adaptive immune cells (like T cells and B cells) are present in the cellular infiltration of the cerebral embolism area. Neutrophils are one of the earliest cells to infiltrate the injured brain. Following cerebral ischemia, the substantial release of various cytokines, chemokines and damage-associated molecular patterns (DAMPs) attributes to the activation and recruitment of neutrophils from the bone marrow, spleen, and peripheral circulation to the site of damage (**Table 2**). This process entails the adhesion of the peripheral immune cells to endothelial cells, which facilitates their translocation across the blood-brain barrier, ultimately resulting in barrier disruption, cerebral edema, and brain injury, which indirectly promote the infiltration of peripheral immune cells (69). The number of infiltrating neutrophils peaks on day 3 after cerebral infarction, persists for at least a week, before gradually declining (70). In contrast, T cells preferentially migrate to the lesion boundary, with a marked increase in a few days after ischemia, reaching a peak within one week and persisting for months (71). CD8^+^ cytotoxic T cells are the first T cell subset to infiltrate the infarct lesion within hours after stroke, whereas CD4^+^ and natural killer T cells appear at approximately 24 hours after the onset of ischemia (66). In rodent models of stroke, the number of CD3+ T cells reached a peak on day 7 after permanent middle cerebral artery occlusion (pMCAO), followed by a significant proliferative phase (65). Even on day 14 and 28 after pMCAO, the number of CD3^+^ T cells significantly increased in the ipsilateral brain, not only in the distal core but also in the corpus callosum (72). In contrast, Treg cells require a considerable period of time, spanning several days, to invade the brain following the onset of ischemia, and maintain in a significant number for more than one month (71). In total, T cell subsets exhibited a long-term activation state in experimental ischemic stroke, which suggests a potential harmful role in the early phase and a protective effect in the later phase (73).

**Table 2:** The infiltrating situation of different subtypes of immune cells after the onset of ischemic stroke.

Immunophenotyping of effector cells	Publication date	The infiltrating situation of Immune cell subtypes
Microglia	2015	After the onset of stroke, microglia are immediately activated. Microglial activity involved in tissue damage peaks within 3 to 5 days. (63)
Astrocytes	2023	Astrocytes are activated several minutes after stroke. (64)
CD8^+^ T cells	2021	CD8+ cytotoxic T cells are the first T cell subset to infiltrate the infarct lesion within hours after stroke. (65)
CD4^+^ T cells	2021	CD4+ T cells appear at approximately 24 hours after ischemia. (65)
CD3^+^ T cells	2021	The count of CD3+ T cells reached its highest level on the seventh day following pMCAO. (65)
Neutrophils	2014	After pMCAO (permanent middle cerebral artery occlusion), neutrophils appear in the pia mater and brain parenchyma within 6 and 12 hours, respectively. Neutrophil recruitment peaks within 1 to 3 days and then gradually decreases, reaching a peak between 3 to 5 days after tMCAO. (66)
Dendritic cells	2010	DCs accumulated in the ischemic hemisphere within 24 hours after MCAO reperfusion, particularly in the infarct border region where T lymphocytes gathered, and this accumulation persisted for at least 7 days. (67)
Monocytes/macrophage	2012	Macrophages are rarely detected within the first 48 hours, Their level gradually increases, with a peak during the first week after stroke. (68)

CD4^+^ T cells contribute to the exacerbation of cerebral inflammation and the induction of neuronal death (74, 75). The depletion of CD4^+^ or CD8^+^ T cells in mice subjected to transient MCAO was found to effectively reduce neuronal apoptosis, decrease infarct volume, and promote neurogenesis (76). The removal of CD25^+^ T cells (including Treg cells) was found to have a partial inhibitory effect on neurogenesis and hinder the repair of neural function (77). Treg cells primarily prevented secondary brain injury by coordinating lymphocyte and microglia infiltration in ischemic stroke through IL-10 signaling (78, 79). Treg cells could not only interact with microglia (75), but also regulate their polarization from M1 to M2 via IL-10 pathway (28, 80, 81). The aforementioned studies also demonstrated that Treg cells could not only mitigate neurological damage, but also regulate the steady-state of peripheral immune responses, including the correction of immune suppression and the reduction of peripheral inflammation (82). Conversely, M2 microglia promoted nerve regeneration through two pathways: (1) microglia could secrete an insulin-like growth factor (IGF-1), which increased the proliferation of neural precursor cells (NPCs); (2) M2 microglia regulated immunity and the balance between oligodendrogenesis and neurogenesis by secreting IL-4 and IL-10 (83, 84). The abovementioned content on ischemic stroke has constructed a potential concept of "T cell-microglia-neuron/NPCs" axis (60). We can explain the potential mechanism of regulating immunological function and nerve regeneration after ischemic stroke based on this concept.

### 5.2 Regulation of T cell subtypes CD4/CD8 and Treg cells by GLP-1RA

**Figure 3:**
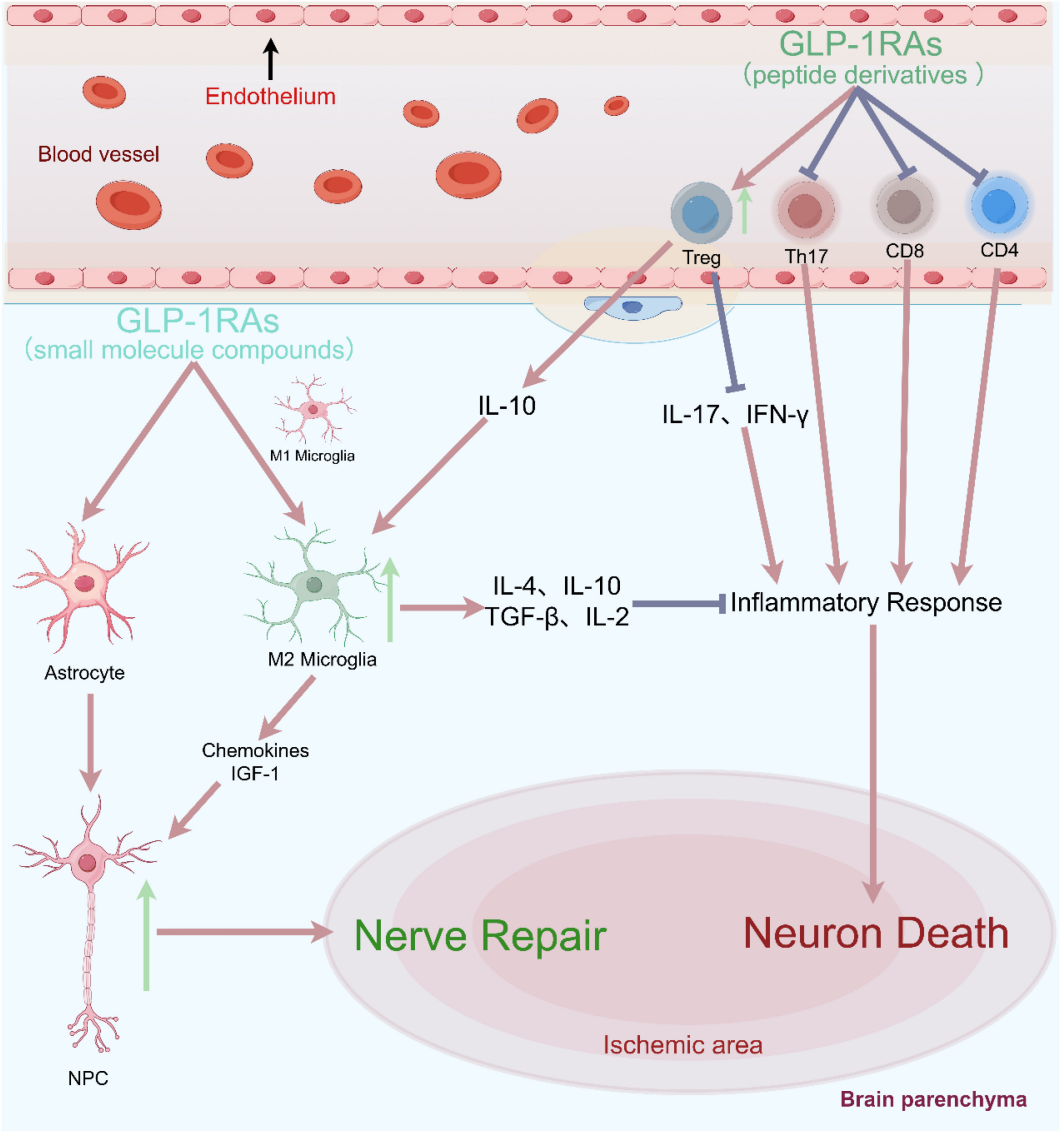
Blocking the recruitment of peripheral effector T cells could significantly reduce neuronal damage and improve clinical prognostic indicators in cerebral ischemia.

GLP-1R is expressed in various immune cell populations of NOD mice, with a higher proportion of mature CD4^+^ and CD8^+^ T cells (approximately 5%-6%). Following T-cell activation GLP-1R expression increased significantly, particularly in CD8^+^ T cells (85). Male Glp1r^−/−^ mice could induce a significant increase in peripheral lymphocytes, but significantly reduced the percentage of CD4^+^CD25^+^FOXP3^+^ Tregs, and had no effect on cell apoptosis and migration (86). T helper cell 17 (Th17) and regulatory T cells (Treg) are two immune cells with opposing functions. Th17 cells promote inflammatory responses, whereas Treg cells inhibit inflammation and maintain peripheral tolerance. Exenatide could promote the increase of Treg cells in mice, but inhibit the tissue infiltration of Th17 cells, suggesting that exenatide exerts immunomodulatory effects by correcting Th17/Treg imbalance (87). The PI3K/Akt/FOXO1 pathway might be involved in the regulation of Th17/Treg balance by exenatide, while FOXO1 inhibitors could block the regulatory effect of exenatide on Th17/Treg balance. In the NTS model, Glp1r^−/−^ mice exhibited immunomodulatory effects, increasing tissue infiltration of neutrophils and T cells. The Th17 marker gene *Rorγt* and the Th1 and Th2 regulatory genes *Tbet* and *Gata3* were also significantly elevated, accompanied by a significant increase in various systemic inflammation-related genes such as interferon gamma (*IFN-γ*), etc (88). *In vitro* experiments showed that liraglutide could reduce the proliferation activity of CD4^+^IFN-γ^+^ T cells (Th1) and CD4^+^IL-17a^+^ T cells (Th17), but increase the number of CD4^+^IL-4^+^ T cells (Th2) and CD4^+^CD25^+^ T cells (Treg). And this regulatory effect was ineffective in Glp1r^−/−^ mice, indicating that the immunoregulatory and anti-inflammatory effects of liraglutide depended on the GLP-1R signaling pathway (89). Besides, GLP-1RA NLY01 could significantly reduce the proportion of infiltrating white blood cells (CD45^high^) and monocytes (Clec12a^+^) in the central nervous system (CNS), while decreasing the number of effector/memory T cells (CD4^+^CD44^+^) and inhibiting inflammation in peripheral circulation and CNS (90). In summary, GLP-1RA could reduce the proliferation of effector T cells, promote the generation of Treg cells, and enhance the immunosuppressive function of Treg cells (**Figure 3**) (91).

Early immune cell infiltration in rodent stroke is dominated by neutrophils and macrophages/microglia, whereas in human ischemic stroke it is different, consisting mainly of neutrophils and T cells (92). In clinical practice, it was also found that immune activation led to a rapid and significant upregulation of GLP-1R expression in naive CD4^+^ T cells enriched more than 40-fold from the human body (93). Among different polarization conditions (including Th1, Th2, Th17, and Treg polarization conditions), the expression level of GLP-1R was highest in CD4^+^ T cells under Treg polarization conditions, and the proportion of GLP-1R^+^ cells could reach 29-34%. These GLP-1R^+^ cells were often accompanied by high expression of Foxp3 and CD25 and low expression of IL-7Rα (CD127), which were characteristic protein markers of Treg cells. Therefore, this study provided the first evidence of functional GLP-1R expression in human iTreg cells, and suggested that GLP-1R might play a key role in the anti-inflammatory effects attributed to GLP-1RA. *In vitro* cell therapy also indicated that infusion of Treg cells could increase the expression of anti-inflammatory factors (such as IL-10, IL-4, TGF-β, IL-2), while reducing the levels of pro-inflammatory factors (such as IL-17, IFN-γ) (94). We speculated that GLP-1RA might regulate Treg cells with high expression of GLP-1R after activation, and exert anti-inflammatory effects by increasing anti-inflammatory factors such as IL-10.

### 5.3 The "Tregs-Microglia-Neuron/NPCs" axis and GLP-1R/cAMP/PKA/IL-10 mediated immunosuppressive and neuroprotective effects of GLP-1RAs in ischemic stroke

In summary, in the pathological state of ischemic stroke, T cells preferentially migrated to the lesion boundary, and significantly increased after ischemia, peaking in the following weeks and persisting for several months (71). CD8^+^ cytotoxic T cells are the first subpopulation of T cells that invade the ischemic brain within a few hours after stroke (66), while Treg cells take many days to infiltrate the brain after the onset of ischemia and remain in significant numbers for more than one month (71). Neuroinflammation after cerebral ischemia is the combined result of the activated resident microglia and infiltrating peripheral leukocytes. Despite the huge difference in their numbers, with only a few thousand lymphocytes invading the brain and more than 50 times higher numbers of resident microglia, microglia could amplify the impact of T cells on the cerebral immune environment, making T cells the largest subtype of white blood cells contributing to secondary inflammatory damage after ischemia (70). In lymphocyte-deficient mice (T and B cells), the number of activated microglia was significantly reduced after ischemic stroke, particularly in the ischemic border zone (95). Furthermore, blocking the recruitment of peripheral effector T cells could significantly reduce neuronal damage and improve clinical prognostic indicators in different cerebral ischemias (**Figure 3**) (76, 96). Therefore, we speculated that T cells could induce microglial polarization through direct contact, cytokine-mediated communication or antigen presentation, thereby amplifying their impact on the cerebral immune milieu.

Further studies showed that different subtypes of T cells had apparently different effects on microglia. Differentiated Th1, Tregs, and IL-10 overexpressing engineered T cells were separately injected into the cerebellar medullary cistern of lymphocyte deficient (Rag1^−/−^) mice after 24 hours in MCAO-induced insults. The results showed that Th1 cells polarized microglia towards high expression of INF response related genes (*Irf7* and *Stat1*), which further exacerbated the immune response in the later stages of neurodegenerative diseases. In contrast, single injection of Tregs or IL-10 overexpressing engineered T cells promoted gene expression of chemokines/cytokines in microglia (Ccl2, Ccl7, and Cxcl10), regulated the chemotactic behavior of microglia and neural stem cells, promoted angiogenesis, and was speculated to contribute to the repair of ischemic injury (95). Another study also demonstrated that depletion of Treg cells could increase the area of delayed cerebral infarction and further exacerbate neurofunctional damage (78). The absence of Treg cells enhanced the activation of resident and invading inflammatory cells (including microglia and T cells) after ischemia, which were the main sources of harmful TNF-α and IFN-γ, respectively. In addition, IL-10 could counteract the overexpression of cytokines and delayed brain injury caused by Treg cell deficiency. Similarly, IL-10-deficient Treg cells could not ameliorate ischemic injury, indicating that IL-10 signaling plays a crucial role in immune suppression and neuroprotection of Treg cells. The above evidence suggests that crosstalk between microglia and Tregs is a key determinant of neuronal regeneration and the repair of synaptic plasticity after brain injury (97).

On one hand, GLP-1RA could reduce the proliferation of effector T cells, promote the generation and function of Treg cells. Treg cells could not only suppress immune inflammation, but also promote neurogenesis and neural repair (77). Treg cells mainly coordinated the infiltration of lymphocytes and microglia in ischemic brain tissue through IL-10 signaling to prevent secondary brain injury (78, 79). Treg cells not only interacted with microglia (75), but also regulated the polarization transformation of microglia from M1 to M2 type (28, 80, 81). On the other hand, *in vitro* experiments demonstrated that GLP-1RA morroniside could act directly on primary microglia, and promote the mRNA levels of the M2 microglial cell markers Agr1, C206, IL-4, and IL-10. This meant that GLP-1RA could not only indirectly mediate M2 polarization of microglia by regulating Tregs, but also directly bind to GLP-1R on the membrane surface of microglia, completing M2 polarization through the GLP-1R/cAMP/PKA/IL-10 signaling pathway (28).

Recent research showed that GLP-1RA could reduce plasma TNF-α levels induced by different Toll-like receptor agonists and significantly inhibit neuroinflammation (98). There was no difference in the anti-inflammatory effect of GLP-1RA between *Glp1r* ^Tie2+/+^and *Glp1r* ^Tie2-/-^ mice, indicating that this activity was not dependent on the blood system or endothelial cells. In contrast, the anti-inflammatory effect of GLP-1RA was significantly different between *Glp1r* ^Wnt1+/+^and *Glp1r* ^Wnt1-/-^, *Glp1r* ^Nes+/+^ and *Glp1r* ^Nes-/-^. Wnt1 and Nes were used to label neural crest derived cells and neuroendocrine cells, indicating that this activity required the involvement of central nervous system GLP-1R. These studies suggest that GLP-1RA may inhibit TCR signaling in a GLP-1R-dependent manner, thereby reducing systemic and intestinal inflammation induced by CD8^+^ T cells and other factors (anti-CD3 induction) (99).

In summary, GLP-1RAs have the potential to inhibit neuroinflammation and promote nerve regeneration through the "Tregs-microglia-neuron/NPCs" axis: (1) GLP-1RAs directly and indirectly inhibit the activation of microglia, promote their M2 polarization, and secrete cytokines such as IL-4, IL-10, TGF - β, IL-2 to exert anti-inflammatory effects (100); (2) GLP-1RAs can also induce Tregs and microglia to secrete IL-10, which further promote microglia to secrete chemokines (Ccl2, Ccl7, and Cxcl10) and IGF-1. After stroke, NPCs have the ability to migrate to the lesion site, and chemokines and IGF-1 can promote NPCs proliferation, migration, and differentiation (101, 102). Depletion of CD25-specific antibodies in Treg leads to a decrease in the number of NPCs after experimental stroke (77). Similarly, the increase in the number of Treg cells and the secretion of the key cytokine IL-10 in the lateral ventricle of the ischemic hemisphere are also positively correlated with the increase in NPCs proliferation (103).

## 6 Perspective

Although multiple cytokines (IL-4, IL-10, etc.) together inhibited neuroinflammation in neurological disorders, their functions were significantly different. M2 microglia could be divided into several subtypes such as IL-4-induced M2a and IL-10-induced M2c. The synergistic induction of IL-4 and IL-10 could enhance the expression of M2a-related genes, produce a large amount of CCL24 (eotaxin-2) and promote eosinophil migration (104). In clinical practice focusing on prognostic indicators for ischemic stroke patients found that there was a close negative correlation between IL-10 and NIHSS scores (P=0.0006), but not between IL-4 and NIHSS scores (P=0.088) (105). Comparing the prognosis of ischemic stroke patients between survivors and non survivors, the IL-10 level in the survivors group was significantly higher than that in the non survivors group (P=0.006) (106). Although there is much preclinical evidence as mentioned above, few clinical trials was reported to evaluate the efficacy of GLP-1RAs in the treatment of ischemic stroke or diabetes complicated by ischemic stroke. A letter reported the clinical manifestations of the GLP-1RA exenatide in acute stroke patients for the first time, including treatment regimens and adverse reactions (107). The results showed that 11 patients experienced mild nausea (n=6) and vomiting (n=5), with no serious adverse reactions or deaths. Given the high incidence of vomiting, the author suggested prophylactic administration of antiemetics. The combination of exenatide and antiemetic agents had good safety and tolerability, with no adverse effects on neurological function and prognosis. In addition, we also found that the clinical rationale and protocol design of the TEXAIS (Treatment With Exenatide in Acute Ischemic Stroke) trial (Trial registration: ClinicalTrials.gov/ANZCTR NTA1127 and ACTRN12614001189617) (108, 109). The latest results from the 2023 TEXAIS trial indicated that the use of exenatide (5 µg, twice daily injections for 5 days) did not significantly reduce neurological damage within 7 days in patients with acute ischemic stroke (trial registration numbers: ACTRN12617000409370 and NCT03287076). However, in the exenatide treatment group, the primary outcome rate was 61.2% (n=170), compared to 56.7% in the standard treatment group (n=171), with an adjusted ratio of 1.22 [95% CI, 0.79-1.88] (P=0.38)., which meant that the proportion of subjects who achieved the desired effect in exenatide treatment group was higher (110). GLP-1RAs may be an optimal option for ischemic stroke treatment.

It was noted that no clinical trials had yet been completed on AD, PD, or other neurodegenerative disorders. Thus clinical data on GLP-1 agonists in brain-related conditions remain limited. However, in a double-blinded trial recruiting 54 participants with type 2 diabetes were randomized to liraglutide (1.8 mg/day) or placebo for 26 weeks (111). The mRNA expressions of tumor necrosis factor-α (p = 0.004) and interleukin-1β in peripheral blood mononuclear cells were downregulated (p = 0.046) in the liraglutide-treated group. The clinical evidence of GLP-1RAs in anti-inflammatory effect was also proved in semaglutide, exenatide and others (112–115). Chronic neuroinflammation is considered an important factor in cognitive and memory impairment in neurodegenerative diseases. It was reported that exendin-4 prohibited amyloid-β-induced microglial activation, thereby limiting neuroinflammation, reducing the levels of TNF-α, C1q and IL-1α; and improving recognition (116). Similar activities of other GLP-1 mimetics were also demonstrated in a rodent PD model (117). Another study also indicated that exendin-4 improved memory impairment by dampening the AMPK/NF-κB pathway, reducing levels of IL-1β and TNF-α, and increasing synaptic protein levels (40). Besides, liraglutide could also improve rat memory and cognition function by reduced neuronal apoptosis, tau phosphorylation, and β-site APP cleaving enzyme 1 levels (118). For a long time, scientists believe that chronic neuroinflammation is crucial in cognitive impairment, and that GLP-1RAs protect synaptic and learning functions from neuroinflammation (119, 120).

A genome-wide association study examining common genetic features of AD had identified neuroinflammatory pathways (mainly associated with TNF) to be a key feature of AD, and a driver of risk for these chronic neurodegenerative impairment (121). Plenty of nonsteroidal anti-inflammatory drugs (NSAIDs) were recruited in many clinical trials for AD treatment (122). NSAIDs primarily reduce inflammation by inhibiting cyclooxygenase enzymes (COX-1 and COX-2), which are responsible for the production of prostaglandins, which are involved in the inflammatory response, including the promotion of pain, fever, and tissue damage. Therefore, the anti-inflammatory effect of NSAIDs is mainly applied to peripheral tissues and for short-term inflammation with the limited CNS penetration. Besides, both GLP-1RAs and SGLT2 inhibitors showed the obvious inhibition on neuroinflammation (123, 124). SGLT2 inhibitors could block the reabsorption of glucose in the kidneys, leading to increased glucose excretion and improved glycemic control in type 2 diabetes. The new preclinical evidence also suggested that they might have neuroprotective properties through reducing oxidative stress, modulating inflammatory pathways, and enhancing mitochondrial function (125, 126). However, their effects in humans with brain disorders are still under investigation.

This review was mainly summarized on the basis of research data, and therefore had limitations in many aspects: (1) Although we already knew that GLP-1RA inhibited the activation of microglia and promoted their polarization towards the M2 type, the regulatory mechanism of their induction of polarization in naive CD4^+^ T cells was still unknown, in particular their impact on the proportion of Tregs subtypes in T cells at the sites of infarct lesion. (2) The interaction between microglia and T cells may be bidirectional, as microglia express various molecules involved in antigen presentation and T cell regulation, such as MHC II, CD11c, Dectin-1, etc (127). Do GLP-1RA have a regulatory effect on microglial antigen presentation and microglial-T cell balance? (3) Clinical evidence for GLP-1RA in stroke is still lacking. The latest TEXAIS results in 2023 were negative with a certain trend, which might be limited by the small sample size, short treatment duration and lack of medium to long-term therapeutic evaluation. Current research on GLP-1RA in ischemic stroke should be expanded to include large-scale, long-term follow-up clinical trials to further assess its efficacy and safety. Additionally, evaluating the long-term use of GLP-1RA, particularly across different populations, could provide valuable insights into its therapeutic potential and risk profile, which will contribute to developing more effective treatment strategies and improving the prognosis and quality of life for patients with ischemic stroke and diabetes (128, 129).

Finally, this study elucidated the immunosuppressive and neuroregenerative functions of GLP-1RA based on the "Tregs-microglia-neuron/NPC" axis theory. Given the advantages of small-molecule GLP-1RA, such as rapid diffusion, good BBB permeability and multiple modes of administration, small molecule compounds will be one of the important development trends of GLP-1RA. In the future, we look forward to the clinical research evidence of small-molecule GLP-1RA intervening in ischemic stroke or T2D complicated by ischemic stroke.
